# Editorial: Legacy & emerging contaminants in the aquatic environment—bridging knowledge, policy, and future

**DOI:** 10.3389/ftox.2025.1611852

**Published:** 2025-05-15

**Authors:** Abdul Qadeer, Mengyang Liu, Sanjeeb Mohapatra, Racliffe Weng Seng Lai

**Affiliations:** ^1^ State Key Laboratory of Environmental Criteria and Risk Assessment, National Engineering Laboratory of Lake Pollution Control and Ecological Restoration, State Environmental Protection Key Laboratory for Lake Pollution Control, Chinese Research Academy of Environmental Sciences, Beijing, China; ^2^ O’Neill School of Public and Environmental Affairs, Indiana University, Bloomington, IN, United States; ^3^ Institutue of Soil and Environmental Science, University of Agriculture, Faisalabad, Pakistan; ^4^ State Key Laboratory of Marine Pollution, City University of Hong Kong, Kowloon, Hong Kong SAR, China; ^5^ Department of Water Management, Delft University of Technology, Delft, Netherlands; ^6^ Centre for Regional Oceans and Department of Ocean Science and Technology, Faculty of Science and Technology, University of Macau, Macau, China

**Keywords:** emerging pollutants (EPs), legacy contaminants, environment, lakes, aquatic bodies

## 1 Introduction

Aquatic ecosystems are increasingly under threat from a complex mixture of contaminants, including legacy and emerging. Historically, great focus has been given to legacy pollutants such as polychlorinated biphenyls (PCBs), dichlorodiphenyltrichloroethane (DDT), mercury, and heavy metals—substances known for their environmental persistence, bioaccumulative potential, and long-term ecological and human health impacts (Qadeer et al., 2020; Qadeer et al., 2019; Yang et al., 2019; Wang et al., 2018; Hoskins, 2003). Despite regulatory bans or restrictions in many regions (Gao, 2024; Zimmerman and Anastas, 2015; Qadeer et al., 2022a), these chemicals persist in sediments, biota, and water bodies due to their resistance to degradation, thereby causing lasting ecological disruption (Qadeer et al., 2019; Suedel et al., 1994; Streets et al., 2006; Huang et al., 2022; Coelho et al., 2016).

**FIGURE 1:**
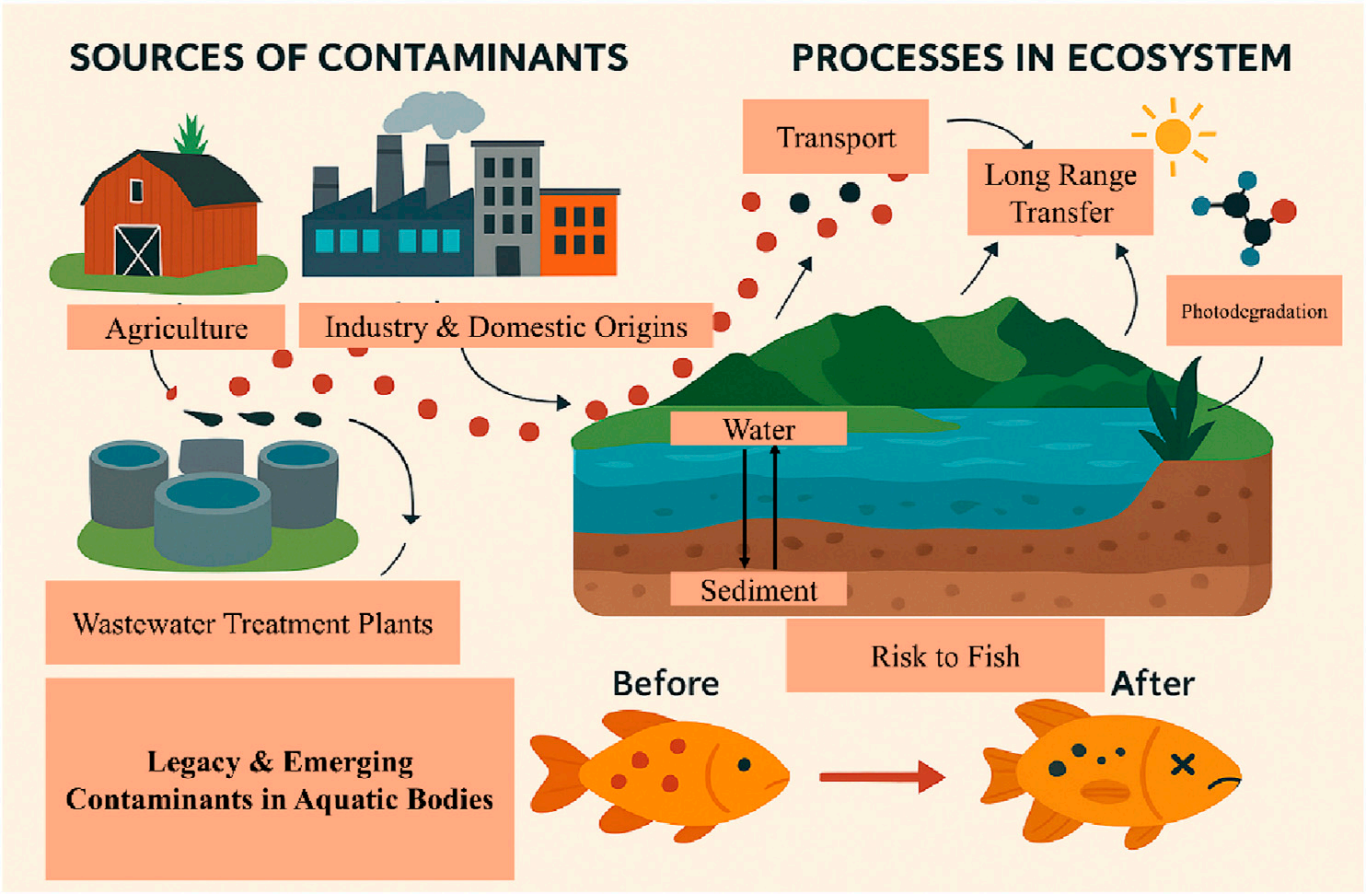
This is a general conceptual illustration of the sources, environmental processes, and ecological risks of legacy and emerging contaminants in aquatic ecosystems.

Concurrently, the scientific community is now contending with a new generation of pollutants, broadly referred to as emerging contaminants. These cover a vast number of chemicals, including but not limited to pharmaceuticals (Katsikaros and Chrysikopoulos, 2021), personal care products (Kachhawaha et al., 2021), microplastics (Biginagwa et al., 2016; Qadeer et al., 2021), plasticizers (Qadeer et al., 2022a; Qadeer et al., 2022b; Zhong et al., 2018), nanomaterials (Noguera-Oviedo and Aga, 2016; Li et al., 2021) and industrial additives such as per- and polyfluoroalkyl substances (PFAS) (Liu et al., 2023; Shen et al., 2018) and flame retardants (Noguera-Oviedo and Aga, 2016). Although these chemicals enter the environment through similar processes as legacy pollutants, they are, in general, more poorly monitored and largely unregulated. Our understanding of their long-term impacts remains limited, but growing evidence suggests they can induce sublethal toxicity, disrupt endocrine systems, impair reproduction, and interact synergistically with other stressors to amplify ecological harm (Qadeer et al., 2021; Zota et al., 2018; Folkerson et al., 2023; Ateia and Scheringer, 2024; Roepke and Sadlier, 2021). A general diagram of their sources, transport and risks of legacy and emerging contaminants is provided in Figure 1.

Together, legacy and emerging contaminants present a dual environmental threat to aquatic systems. Their co-occurrence and chemical mixture interactions necessitate urgent, coordinated efforts to reconsider and reshape traditional monitoring frameworks, risk assessment models, and management strategies. Addressing these interconnected challenges requires not only scientific advancement but also effective integration into environmental policy and on-the-ground practice.

## 2 Overview of the special issue

This Research Topic, “Legacy & Emerging Contaminants in the Aquatic Environment,” features 11 original research articles, offering critical insights into the occurrence, fate, toxicity, and mitigation strategies of both legacy and emerging contaminants across diverse aquatic environments. This collective intellectual property represents efforts from a broad geographic and thematic scope, addressing both long-established and novel pollutants that are gaining increasing scientific and public attention.

Highlights include studies evaluating the impacts of microplastics on marine microbial communities, the toxicological effects of pesticide mixtures on zebrafish embryos, and spatial analyses of phosphorus distribution—linking land use to nutrient runoff. Innovative remediation approaches are also presented, such as the use of hydrothermal charcoals for uranium adsorption and salinity regulation techniques for controlling algal off-flavor compounds in aquaculture systems.

Other contributions examine the presence and distribution of chlorinated paraffins and dechloranes in subarctic lake sediments and biota, the vulnerability of karst aquifers to emerging organic pollutants, and the bioaccumulation of flame retardants in marine fish. A comprehensive assessment of agricultural pesticide residues in European surface waters further emphasizes the complexity of spatial variability and cumulative ecological risks. Collectively, these studies showcase the depth and breadth of current research and underscore the importance of bridging scientific findings with policy development and practical intervention.

## 3 Bridging science with policy and the future

Effectively addressing legacy and emerging contaminants requires not only scientific knowledge, progress, and breakthroughs but also their timely translation into actionable policy and environmental management strategies. Beyond significant advancements in detection techniques, monitoring, and risk assessment models, scientific findings should be more effectively integrated into regulatory frameworks. Bridging this gap calls for interdisciplinary collaboration among scientists, policymakers, industry professionals, and public health authorities. By fostering evidence-based policymaking, encouraging stakeholder engagement, and integrating research into local and international regulations, we can enhance the real-world impact of environmental science and promote sustainable stewardship of aquatic ecosystems.

Although progress has been made, critical knowledge gaps still hinder comprehensive contaminant management. In particular, the effects of contaminant mixtures—often more complex and potent than individual compounds remain poorly understood, especially under realistic environmental conditions. There is also a shortage of long-term monitoring data for many emerging contaminants, limiting our ability to assess chronic exposure risks across trophic levels. Furthermore, the fate, transport, and transformation of emerging pollutants in aquatic systems are still largely uncharted.

To address these gaps, future research must prioritize developing integrated monitoring systems, advanced modeling tools, and high-resolution analytical techniques. Studies should also focus on the interactive and cumulative effects of chemical mixtures to reflect real-world exposure scenarios. Strengthening global research collaboration, harmonizing environmental standards, and promoting open-access data sharing will be essential for accelerating this progress. Additionally, engaging local communities, industry partners, and civil society will enhance the implementation of practical, context-specific solutions to aquatic pollution.
